# Isothermal amplification and rapid detection of *Klebsiella pneumoniae* based on the multiple cross displacement amplification (MCDA) and gold nanoparticle lateral flow biosensor (LFB)

**DOI:** 10.1371/journal.pone.0204332

**Published:** 2018-10-01

**Authors:** Lina Niu, Fan Zhao, Jinlong Chen, Jinqing Nong, Chunmei Wang, Jing Wang, Naishu Gao, Xiaoxue Zhu, Lei Wu, Shoukui Hu

**Affiliations:** 1 Department of Pathogen Biology, School of Basic Medicine and Lifescience, Hainan Medical University, Haikou, Hainan, China; 2 Department of Clinical Laboratory, Peking University Shougang Hospital, Beijing, China; University of Massachusetts Boston, UNITED STATES

## Abstract

*Klebsiella pneumoniae* (*K*. *pneumoniae*) is a frequent pathogen causing nosocomial infections and outbreaks. We developed a multiple cross displacement amplification (MCDA) assay for the detection of *K*. *pneumoniae*, which can get the positive results within 40 minutes’ isothermal amplification. Gold-nanoparticle lateral flow biosensor (LFB) and colorimetric indicators were used for the rapid readouts of MCDA amplification. The detection limit of this assay was 100 fg per reaction at 65°C, which was confirmed to be the optimal amplification temperature according to the real time turbidimeters. For specificity, all of the 30 clinical-source *K*. *pneumoniae* strains were positive for the MCDA, and all of the non-*K*. *pneumoniae* strains belonging to 31 different species were negative for this MCDA assay. To evaluate the practical applicability of this method, we assessed its detection limit for *K*. *pneumoniae* strains in sputum samples (24 CFU per reaction), and DNA templates of 100 sputum samples further underwent the MCDA-LFB tests. All of the sputum samples being positive for *K*. *pneumoniae* (30/100) with the culture method were successfully identified with the MCDA assay, the detection power of which was higher than that of polymerase chain reaction (PCR) (25/100). Thus, the MCDA test for *K*. *pneumoniae* combined with the gold nanoparticle LFB as the results readout scheme, are simple, specific, and sensitive methods for the rapid diagnosis of *K*. *pneumoniae* in clinical samples.

## Introduction

*Klebsiella pneumoniae* (*K*. *pneumoniae*) is a gram negative opportunistic pathogen belonging to *Klebsiella spp*. *K*. *pneumoniae* mainly attack the immunocompromised populations, manifested as pneumonia, meningitis, bacteremia, sepsis, urinary tract infection and focal infections [1,2]. *K*. *pneumoniae* is widely present in the hospital environment of hospitals, even in the medical equipment like bronchoscopy, duodenoscopy, gastroscopy and more [2–4]. Moreover, it can colonize in the gastrointestinal tract asymptomatically and on the hands of hospitalized patients or the hospital personnels, serving as reservoirs and carriers for this conditioned pathogen [5]. Thus, *K*. *pneumoniae* is one common reason for the nosocomial infections, especially in the intensive care units [6]. In the past two decades, multi-drug resistance of *K*. *pneumoniae* has increased substantially, which offer advantages over other antimicrobial-sensitive bacteria in the hospital and patients [6]. *K*. *pneumoniae* often contaminates hospital equipment and hospitalized patients who have shared the contaminated equipment; therefore, nosocomial outbreaks caused by *K*. *pneumoniae* have been frequently reported among the hospitalized patients [3,4,7]. Thus, prophylaxis measures, especially for sensitive and rapid diagnostic assays, are required for the control of healthcare-associated infections caused by *K*. *pneumoniae*.

Multiple cross displacement amplification (MCDA) was a nucleotide-based isothermal amplification assay devised by Wang *et al*. (Patent number CN201510280765.X) [8]. This method has superior performance compared to traditional polymerase chain reaction (PCR). Rapidity and simplicity in operation are the outstanding features of MCDA, which merely requires for a thermostatic environment (ranging from 60°C to 68°C) of less than 40 minutes for amplification [8]. These characteristics will fit in well with the field investigations especially when experimental settings are limited. Besides, the high concentration of primers in the reaction system allows for a high sensitivity of this method, the detection limit of which can reach 10 fg/μL [9]. Moreover, MCDA is economic friendly with each reaction system costs an average of $3.5, making it possible for the widely application in the poor-resource settings [10].

There were many techniques developed for the verification of positive amplification [10]. Gel electrophoresis and imaging is the traditional method being widely used, but it is laborious, time consuming and equipment demanding. Real-time fluorescent instruments and real-time turbidimeters are quick and quantitative, but the apparatuses are expensive and unportable, which limit their application in poor-source settings and field investigations. Colorimetric indicators were quick and convenient, but the colorimetric indicators were subjective and sometimes may be difficult to determine the results [11]. The gold nanoparticle lateral flow biosensor (LFB) is a novel method to detect specific DNA fragments, which is based on the binding of antibodies (embedded on the LFB) and haptens (labeled on the 5ʹ side of primers) [12–14]. Recently, LFB was used for reporting MCDA results [9–11,15]. When there were positive amplification products, the amplified sequences labeled with haptens will be bound to the antibodies embedded on the test line, showing the red color. In comparison with other method for indicating MCDA results, LFB is relatively simple, quick and objective.

Here, we developed a MCDA detection assay for *K*. *pneumonia*, which is a common cause for the nosocomial infections. The optimal amplification temperature was screened. Sensitivity, specificity and the clinical application of the *K*. *pneumoniae*-MCDA assay were assessed. LFB and colorimetric indicators were applied as the main methods for the identification of positive amplification products, which were confirmed with turbidimeters and gel electrophoresis [9,11].

## Materials and methods

### Design of MCDA primers

**Fig 1:**
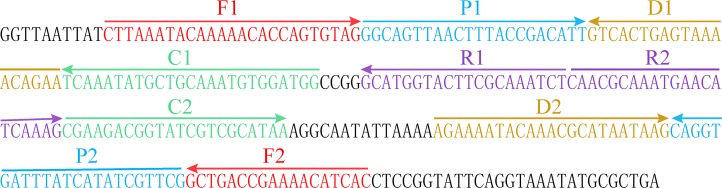
The location of the five pairs of primers on the *rcsA* gene. Different colors represented different pairs of primers. The direction of the arrowed lines indicated the sequences from 5ʹ to 3ʹ ends.

The five pairs of primers for the isothermal amplification and identification of *K*. *pneumoniae* were designed according to the principle of MCDA developed by Wang *et al*. [9,11]. Two widely used softwares (PrimerExplorer V4 and Primer Primer 5.0) were used to design the MCDA primers on the *rcsA* gene, which was specific for *K*. *pneumoniae* and had been used for the genome-based identification of this pathogen (GenBank accession No. NC_012731.1) [16]. Specificity of the primers designed was verified with the nucleotide BLAST (Basic Local Alignment Search Tool) of the GenBank database. The location and the sequence of each primer were indicated in Fig 1 and Table 1, respectively. Moreover, the 5ʹ- ends of the primers C1 and D1 were labeled with biotin and fluorescein isothiocyanate (FITC), respectively. All oligomers were synthesized by Ruiboxingke. C., Ltd. (Beijing, China).

**Table 1:** Primers of the MCDA assay for the identification of *K*. *pneumoniae*.

Primers^a^	Sequences and modifications (5ʹ-3ʹ)	Length^b^
**F1**	CTTAAATACAAAAACACCAGTGTAG	25 nt
**CP1**	CCATCCACATTTGCAGCATATTTGAGGCAGTTAACTTTACCGACATT	47 mer
**C1**	CCATCCACATTTGCAGCATATTTGA	25 nt
C1*	Biotin-CCATCCACATTTGCAGCATATTTGA	25 nt
**D1**	TTCTGTTTTACTCAGTGAC	19 nt
D1*	FITC-TTCTGTTTTACTCAGTGAC	19 nt
**R1**	AGATTTGCGAAGTACCATGC	20 nt
**R2**	CAACGCAAATGAACATCAAAG	21 nt
**D2**	AGAAAATACAAACGCATAATAAG	23 nt
**C2**	CGAAGACGGTATCGTCGCATAA	22 nt
**CP2**	CGAAGACGGTATCGTCGCATAACGAACGATATGATAAATCACCTG	45 mer
**F2**	GTGATGTTTTCGGTCAGC	18 nt

^a^ C1*, 5ʹ-labeled with biotin for the MCDA-LFB assay; D1*, 5ʹ-labeled with FITC for the MCDA-LFB assay.

^b^ nt refers to the nucleotide; mer refers to monomeric.

### The *K*. *pneumoniae*-MCDA-LFB assay

The standard MCDA assay was performed generally in accordance with that of Wang *et al*. [9,11]. It was carried out in a 25 μL reaction system including: 0.4 μM/L each of F1 and F2, 1.6 μM/L each of CP1 and CP2, 0.8 μM/L each of C1 (C1*), C2, R1, R2, D1(D1*) and D2, 12.5 μL 2 × Reaction Buffer, 1 μL *Bst* DNA polymerase (10 U) and 1 μL DNA templates. The reaction buffer and the *Bst* DNA polymerase were obtained from the isothermal amplification kits (Haitaizhengyuan, Beijing, China). Mixtures containing 1 μL genomic DNA of *Staphylococcus aureus* (67.5 ng/μL) and *Salmonella typhii* (130.0 ng/μL) were used as the negative controls. One mixture containing 1 μL of double distilled water was used as the blank control.

**Fig 2:**
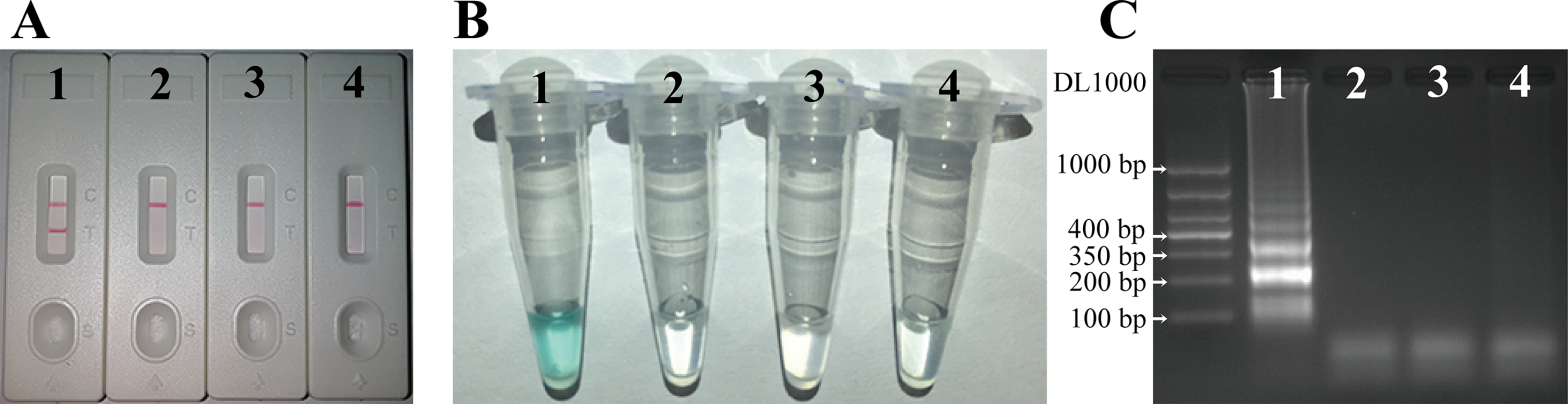
The *K*. *pneumoniae*-MCDA-LFB assay. There were four reactions tested: 1, the reference strain of *K*. *pneumoniae* ATCC2146 (10 pg per reaction); 2, *Staphylococcus aureus* (67.5 ng per reaction); 3, *Salmonella typhii* (130.0 ng per reaction); 4, distilled water. Only the reaction with *K*. *pneumoniae* ATCC2146 showed the positive results: blue in color (Fig 2A), ladder bands by gel electrophoresis (Fig 2B), and the two red lines (both the positive line and the control line) indicated on the LFB (Fig 2C).

The reaction systems were isothermally amplified at 65°C (which was verified to be the optimal amplification temperature with high efficiency), followed by 85°C for 5 minutes to terminate the amplification. Amplicons were read by LFB detection, colorimetric indicators, and gel electrophoresis. Preparation of LFB and detection using the biosensor were as previously described by Wang *et al*. [10,15]. On the LFB, 0.2 μL of the MCDA products followed by 100 μL of the running buffer (0.01 M phosphate-buffered saline, pH 7.4 with 1% Tween 20) were deposited on the sample pad. After 2 minutes, two red lines (the test line and the control line) are observed in positive reactions, but only the control line is visualized for the negative and blank control reactions (Fig 2A). It should be noted that during the LFB reactions, the LFB pads should be tilted with the sample pads being placed lower than the absorbent pads for about 30^o^, which will be in favor of the siphon actions. When using colorimetric indicators, the color of the positive reactions will remain to be blue, while the negative and blank controls will fade into colorless (Fig 2B). In 2% agarose gel electrophoresis, the MCDA amplification products were running at 80 volts for 1 hour. The gel image was captured under ultra violet with the GEL DOC XR (Bio-Rad, America). A ladder of multiple bands was observed for the positive amplifications, but the ladder bands were not observed in the negative and blank controls **(**Fig 2C).

### The optimal amplification temperature

In order to screen the optimal temperature for the MCDA assay, the identical reaction systems (10 pg of ATCC2146) were amplified at temperatures (60°C to 67°C) The real time turbidity of each reaction was recorded, with > 0.1 as the threshold value for the positive reactions at 650 nm of optical density. DNA of *Staphylococcus aureus* (67.5 ng per reaction) was used as the negative control and distilled water as the blank control. The temperature that enables the amplification completed efficiently with high amplification products, were selected as the optimal temperature for the MDCA test of *K*. *pneumoniae* specific gene *rcsA*. The optimal temperature screening experiment was repeated three times. In order to confirm the optimal amplification temperature, we repeated the temperatures of 63°C, 64°C, 65°C and 66°C (which were found to be the potential candidates of the optimal temperature) for another 10 times.

### The sensitivity of *K*. *pneumoniae*-MCDA assay

To assess the sensitivity of the MCDA assay for the identification of *K*. *pneumonia*, the DNA template of ATCC2146 was serially diluted from 10 ng/μL to 0.1 fg/μL with distilled water. Real time turbidity of eight different DNA content (10 ng, 10 pg, 1 pg, 100 fg, 10 fg, 1 fg, 0.1 fg, and distilled water) were measured to identify the limit of detection of the MCDA assay which was empirically ranging from 1 pg to 1 fg according to Wang *et al*.’s [15,17]. DNA templates of *Staphylococcus aureus* and *Salmonella typhii* were used as the negative controls (NC), and distilled water as the blank control (BC). The amplification products were further verified with the colorimetric indicators, gel electrophoresis and the LFB method. The sensitivity evaluation experiment was repeated three times.

Moreover, we evaluated the sensitivity of the MCDA-LFB assay to detect the *K*. *pneumoniae* strains in mixture of other non-*K*. *pneumoniae* strains. We prepared the MCDA reactions containing different concentration of *K*. *pneumoniae* (ranging from 10 ng to 0.1 fg per reaction, and the blank control). Meanwhile, 1 μL DNA templates of one non-*K*. *pneumoniae* strain (i.e. *Staphylococcus aureus* at 67.5 ng/μL, *Salmonella typhii* at 130.0 ng/μL, *Acinetobacter baumannii* at 55.3 ng/μL, and *Streptococcus pneumoniae* at 82.2 ng/μL) was added into the reactions.

### The specificity of *K*. *pneumoniae*-MCDA-LFB assay

To evaluate the specificity of the MCDA assay, the DNA templates of 31 strains of *K*. *pneumoniae* including ATCC2146 and 30 clinical-source *K*. *pneumoniae* strains, as well as 35 non-*K*. *pneumoniae* strains were tested for the MDCA assay. DNA of the pure culture strains were extracted with the DNA extraction kits (Tiangen, Beijing). We initiatively analyzed the amplification products using the colorimetric indicators. Furthermore, the amplification products from 24 strains, including 12 clinical-source *K*. *pneumoniae* strains and 12 non-*K*. *pneumoniae* strains were selected randomly for verification using the LFB test. The specificity assessment was confirmed three times.

### The clinical samples evaluation for the MCDA based identification of *K*. *pneumoniae*

To evaluate the practical application of the MCDA-LFB assay, we assessed its detection limit for *K*. *pneumoniae* strains in sputum samples, by artificially adding the diluted ATCC2146 into the sputum samples. Briefly, one single colony of the ATCC2146 on the Columbia sheep blood agar, was inoculated into 5 ml of the brain heart infusion and incubated at 37°C (220 rpm/min) for about 14 hours. Then, 100 μL of the bacteria suspension was streaked into 5 ml of brain heart infusion and incubated (37°C, 220 rpm/min) until the optical density (OD) at 600 nm reached to 0.6. The bacteria suspension (OD 0.6) was then 10-fold serially diluted (from 10^−1^ to 10^−10^) with 0.01 M phosphate buffered saline, pH7.4. The aliquots of 100 μL dilutions (10^−5^ to 10^−7^) were spread in triplicate onto the Columbia blood plate medium agars and incubated at 37°C. The number of colony forming units (CFU) was counted after 24 hours’ culture. Meanwhile, aliquots of 100 μL diluted suspensions were added into the sputum samples, which have been confirmed to be negative for this pathogen with the culture method in advance. The concentration of the ATCC2146 was adjusted to 2.4 × 10^0^, 2.4 × 10^1^, 2.4 × 10^2^, 2.4 × 10^3^, 2.4 × 10^4^, 2.4 × 10^5^, 2.4 × 10^6^ CFU/ml in the sputum samples. DNA templates of the artificially contaminated sputum samples (100 μL) were extracted and dissolved in 10 μL of Qiagen elution buffer (Qiagen, Germany). Then, 1 μL of the DNA templates were tested with both the MCDA-LFB. And the PCR method was used as comparison, the primers of which were indicated in S1 Table. The limit of detection (LOD) evaluation assay in the sputum samples was performed triplicate independently.

Moreover, 100 human sputum samples from the Department of Clinical Laboratory, Peking University Shougang Hospital, were collected and extracted for the DNA with the DNA Microbiome Kits (Qiagen, Germany) since March 2017 to June 2017. All patients who provided samples gave written informed consent. The clinical laboratory of Shougang Hospital has access to the information that could identify individual participants during the samples collection. This study was approved by the Ethics Committee of Shougang Hospital, and conducted according to the medical research regulations of the Ministry of Health, China. These samples were previously identified by the culture and biochemical method (the reference method) and stored at −70°C until use in the MCDA-LFB assay. By the reference method, 30 (30%) of the 100 samples were identified as *K*. *pneumoniae*-positive, while the remainders were identified as containing other bacteria or negative. The other bacteria included *Acinetobacter baumannii*, *Staphylococcus aureus*, *Pseudomonas aeruginosa* and *Staphylococcus hominis* with the culture methods. DNA templates of the sputum samples were tested with the MCDA-LFB assay by adding 1 μL of the DNA templates of the sputum samples. In addition, the PCR amplification test for *K*. *pneumoniae* was performed to compare with the results of MCDA-LFB test and the reference method.

### Statistical analysis

Sensitivity of the MCDA-LFB assay or PCR for clinical sample detection was computed by Number of true positive/ (Number of true positive + Number of false negative) ×100%. Specificity of the MCDA-LFB assay or PCR for clinical detection was computed by Number of true negative/ (Number of true negative + Number of false positive) ×100%. The receiver-operating characteristic (ROC) curves analysis were performed in detection result for the 100 clinical samples by MCDA-LFB assay or PCR. Statistical Program for Social Sciences (SPSS) software version 20.0 (Inc., Chicago, IL, USA) was used for computation.

## Results

### The MCDA assay and the LFB verification for *K*. *pneumoniae*

We found one of the three sets of primers designed was applicable for the identification of *K*. *pneumoniae* with the negative controls and the blank control being negative using the three monitoring methods. For the positive reactions, there were two red bands on the LFBs (Fig 2A), the color of the reaction remain to be blue (Fig 2B), and the electrophoresis gel showed ladder bands (Fig 2C). As there were five pairs of primers working on the target sequence at different binding sites [8], the MCDA reactions produced amplification products of different sizes showing ladder bands in the electrophoresis gel (Fig 2C). During the isothermal amplification process, the cross primers of CP1 and CP2 produced amplification products of increasing sizes as long as 1000 bp or more. For the negative reactions, there was only one red band on the control line; the reactions faded into colorless; and there were no ladder bands as large as 1000 bp by electrophoresis.

### The optimal amplification temperature

**Fig 3:**
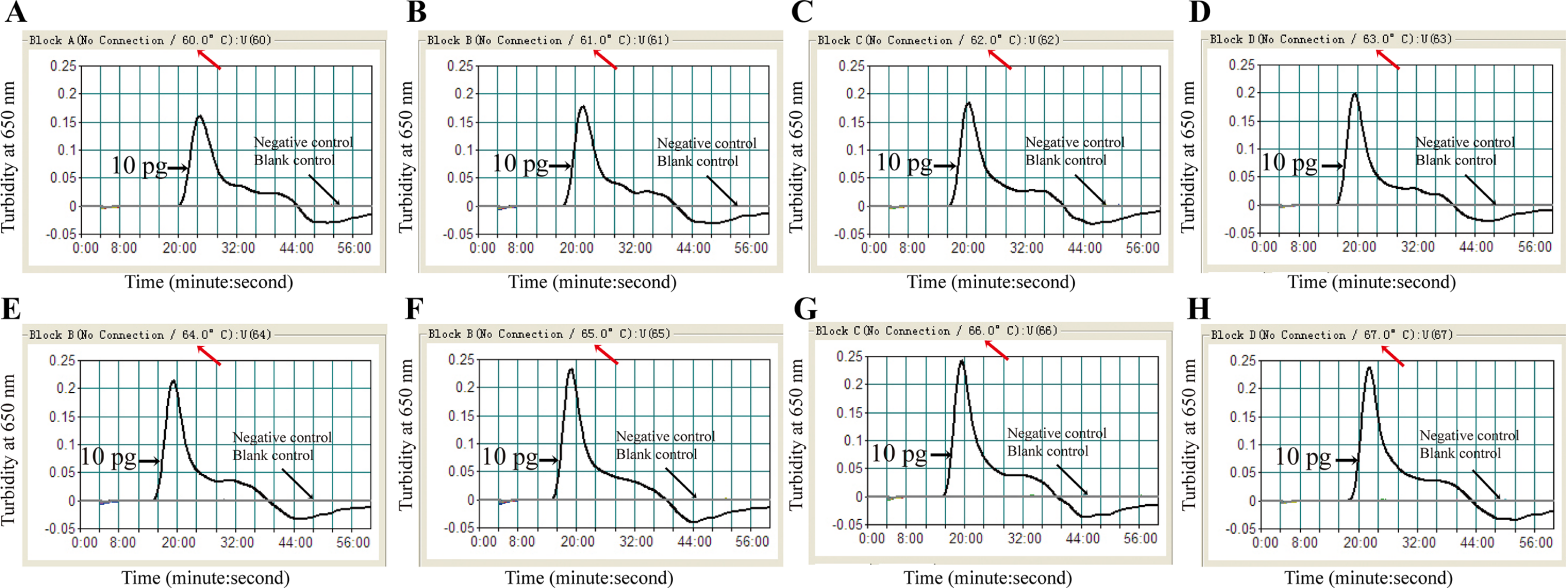
Screening the optimum temperature for the *K*. *pneumoniae* MCDA assay. DNA templates of ATCC2146 (10 pg) were amplified under different temperatures (60 to 67°C), and their real time turbidities were recorded using a turbidimeter at 650 nm (A–H). DNA of *Staphylococcus aureus* was used as the negative control, and distilled water was the blank control. MCDA, multiple cross displacement amplification.

We identified the optimal amplification temperature (ranging from 60°C to 67°C of the *K*. *pneumoniae*-MCDA assay). We found that 65°C was the best candidate, the turbidity of which occurred earlier than the other temperatures with amplification products being relatively higher (Fig 3). In order to confirm the optimal amplification temperature, we repeated the temperatures of 63°C, 64°C, 65°C and 66°C for another 10 times, with the real time turbidity recorded. There were 9 times indicated that 65°C was the optimal temperature, the turbidity of which occurred earlier than the other temperatures with amplification products being relatively higher. And there was one time that no obvious difference was observed among the turbidity lines of the four temperatures. It suggested that 65°C was the optimal amplification temperature.

### The sensitivity of the *K*. *pneumoniae*-MCDA-LFB assay

**Fig 4:**
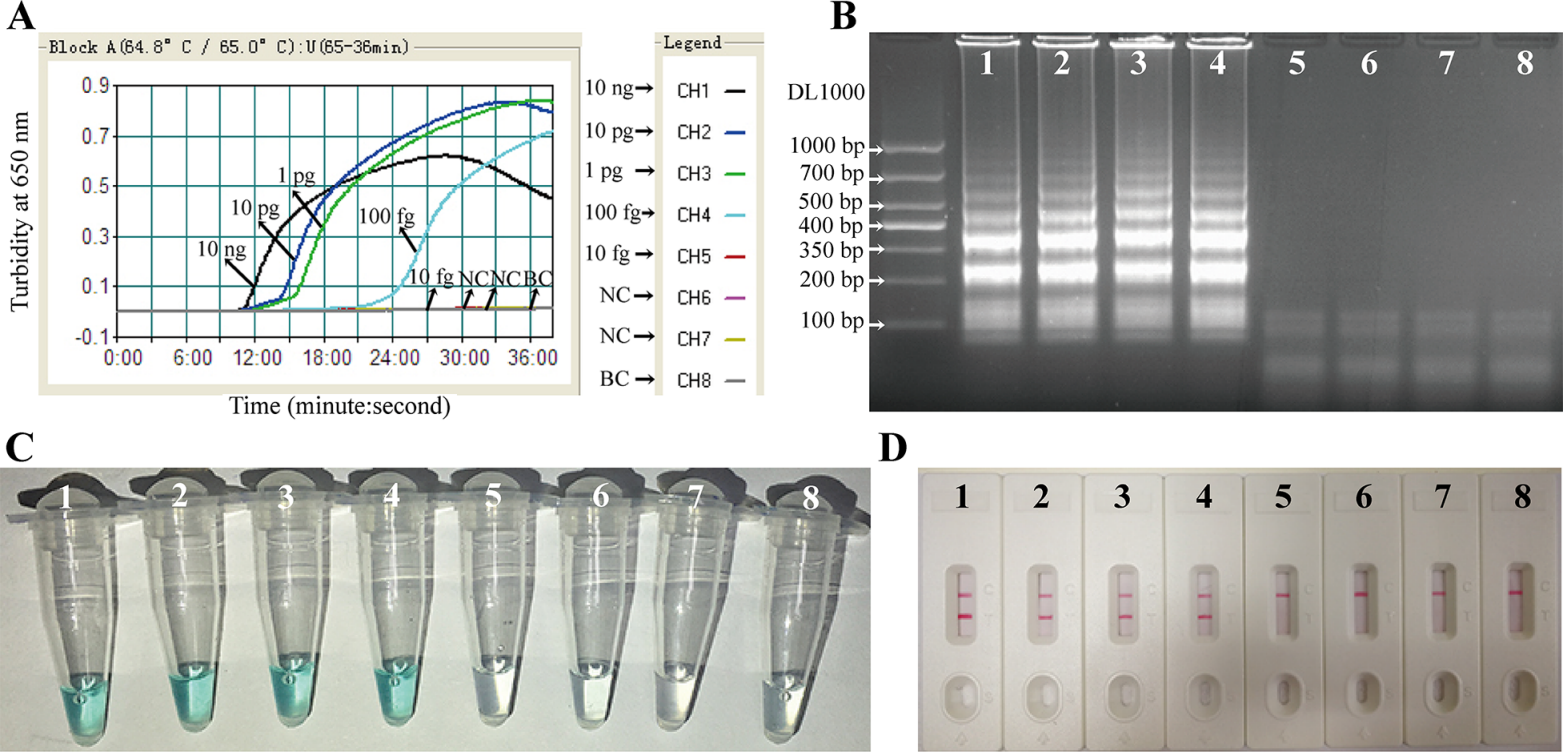
Sensitivity of the *K*. *pneumoniae-*MCDA assay. DNA template of ATCC2146 at different concentrations (10 ng/μL, 10 pg/μL, 1 pg/μL, 100 fg/μL, 10 fg/μL), were amplified at 65°C. DNA of *Staphylococcus aureus* and *Salmonella typhii* were used as the negative controls (NC) and distilled water as the blank control (BC). (**A**) The real time turbidity lines of the eight reactions using the turbidimeter. The threshold value for the positive reactions was > 0.1 by the real time turbidimeter at an optical density of 650 nm. (B) The electrophoresis analysis of the MCDA products separated by a 2% agarose gel (stained by ethidium bromide). (C) The colorimetric indicators of the positive and negative amplifications. (D) The LFB analysis of the amplification products. MCDA, multiple cross displacement amplification; LFB, gold nanoparticle lateral flow biosensor.

By measuring the turbidity of the reactions with different DNA content (10 ng, 10 pg, 1 pg, 100 fg, 10 fg), the detection limit of the *K*. *pneumonia*-MCDA assay was 100 fg (Fig 4A). The results were in accordance with that of colorimetric indicators, gel electrophoresis and LFB results (Fig 4).

We also assessed the sensitivity of the *K*. *pneumoniae*-MCDA-LFB assay when in mixture of other non-K. pneumoniae strains, by 1 μL DNA of non-*K*. *pneumoniae* strain (*Staphylococcus aureus* at 67.5 ng/μL, *Salmonella typhii* at 130.0 ng/μL, *Acinetobacter baumannii* at 55.3 ng/μL, and *Streptococcus pneumoniae* at 82.2 ng/μL) into 8 reactions containing different concentration of ATCC2146 (10 ng, 10 pg, 1 pg, 100 fg, 10 fg, 1 fg, 0.1 fg and distilled water). The LOD for the *K*. *pneumoniae*-MCDA assay remained to be 100 fg (S1 Fig).

### The specificity of the *K*. *pneumoniae*-MCDA-LFB assay

**Fig 5:**
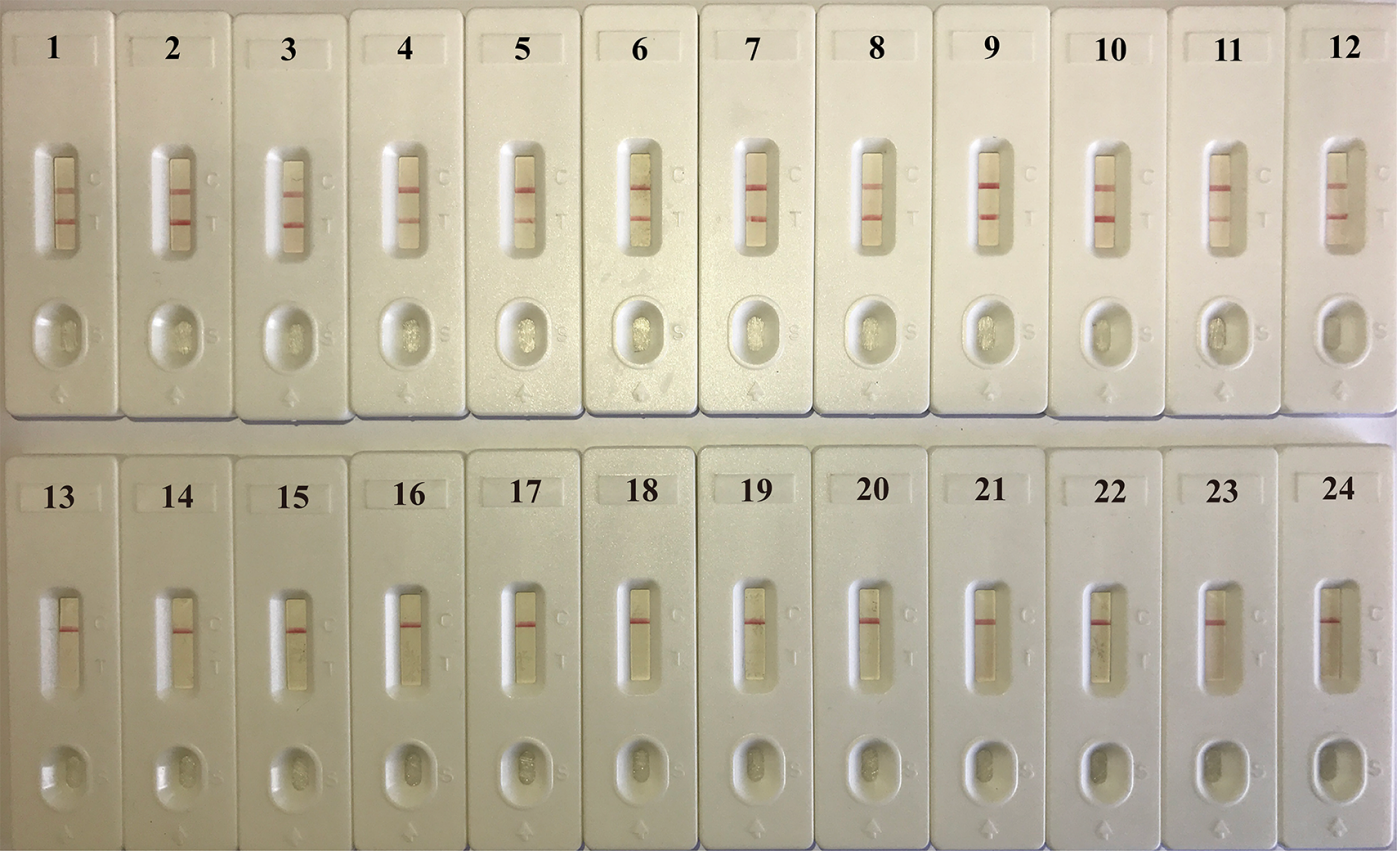
Specificity of the *K*. *pneumoniae*-MCDA-LFB assay. Biosensor 1, *K*. *pneumoniae* (ATCC2146). Biosensors 2–6, different *K*. *pneumoniae* strains from sputum. Biosensors 7–8, different *K*. *pneumoniae* strains from urine. Biosensors 9–12, *K*. *pneumoniae* strains from blood, pharyngeal swabs, puncture fluid, and wounds, respectively. Biosensors 13–24, *Aeromonas caviae*, *Bacillus cereus*, *Citrobacter braakii*, *Corynebacterium stationis*, *Enterobacter sakazakii*, *Enterotoxigenic Escherichia coli*, *Listeria monocytogenes*, *Salmonella enterica*, *Shigella flexneri*, *Staphylococcus aureus*, *Streptococcus pneumoniae*, and *Streptococcus suis*. MCDA, multiple cross displacement amplification; LFB, gold nanoparticle lateral flow biosensor.

By testing the 30 clinical-source *K*. *pneumoniae* strains and 35 non-*K*. *pneumoniae* strains, all of the *K*. *pneumoniae* strains showed positive results with the color remained to be blue, while all of the 35 non-*K*. *pneumoniae* reactions faded into colorless after the amplification (Table 2). When using the LFB to test the amplification products of the randomly selected 24 strains for validation, the results are in accordance with those using the colorimetric indicators. For the LFBs of the *K*. *pneumoniae* strains, there were two lines on the LFB pads, including the test line and the control line. For the non-*K*. *pneumoniae* strains of different species, there was only one red mark in the control line on the LFB pads (Fig 5). Furthermore, the MCDA-LFB results of the *K*. *pneumoniae* and non-*K*. *pneumoniae* strains were the same in the triplicate repeats.

**Table 2:** Bacteria strains used in the *K*. *pneumoniae*-MCDA assay and the specificity evaluation results.

ID	Strains	Sources	MCDA assay result ^a^	Selected for LFB validation (n)
1	*Klebsiella pneumoniae*	ATCC2146	P	1
2–17	*Klebsiella pneumoniae*	Isolated strains sputum	P	5
18–25	*Klebsiella pneumoniae*	Isolated strains from urine	P	2
26–27	*Klebsiella pneumoniae*	Isolated strains from blood	P	1
28–29	*Klebsiella pneumoniae*	Isolated strains from pharyngeal swabs	P	1
30	*Klebsiella pneumoniae*	Isolated strains from puncture fluid	P	1
31	*Klebsiella pneumoniae*	Isolated strains from a wound	P	1
32	*Acinetobacter baumannii*	Isolated strains from sputum	N	0
33	*Aeromonas caviae*	Isolated strains (U) ^b^	N	1
34	*Aeromonas sobria*	Isolated strains (U)	N	0
35	*Bacillus cereus*	Isolated strains (U)	N	1
36	*Citrobacter braakii*	Isolated strains (U)	N	1
37	*Citrobacter freundii*	Isolated strains (U)	N	0
38	*Citrobacter youngae*	Isolated strains (U)	N	0
39	*Corynebacterium stationis*	Isolated strains from the environment	N	1
40	*Corynebacterium ammoniagenes*	Isolated strains from the environment	N	0
41	*Enterobacter sakazakii *	Isolated strains from feces	N	1
42	*Enteroaggregative E*. *coli (EAEC)*	Isolated strains from feces	N	0
43	*Enteroinvasive Escherichia coli (EIEC)*	Isolated strains from feces	N	0
44	*Enteropathogen Escherichia coli (EPEC)*	Isolated strains from feces	N	0
45	*Enterotoxigenic Escherichia coli (ETEC)*	Isolated strains from feces	N	1
46	*Shigatoxin-producing E*. *coli (STEC)*	Isolated strains from feces	N	0
47	*Listeria ivanovii*	Isolated strains (U)	N	0
48	*Listeria monocytogenes*	Isolated strains from blood	N	1
49–51	*Pseudomonas aeruginosa*	Isolated strains from sputum	N	0
52	*Proteus mirabilis*	Isolated strains from the environment	N	0
53	*Providencia rettgeri*	Isolated strains from the environment	N	0
54	*Salmonella enteric*	Isolated strains from feces	N	1
55	*Salmonella typhi*	Isolated strains from feces	N	0
56	*Shigella dysenteriae*	Isolated strains from feces	N	0
57	*Shigella flexneri*	Isolated strains from feces	N	1
58	*Shigella sonnei*	Isolated strains from feces	N	0
59	*Staphylococcus cohnii*	Isolated strains from the environment	N	0
60	*Staphylococcus sciuri*	Isolated strains from the environment	N	0
61	*Staphylococcus aureus*	Isolated strains from sputum	N	1
62	*Staphylococcus epidermidis*	Isolated strains (U)	N	0
63	*Staphylococcus saprophyticus*	Isolated strains (U)	N	0
64	*Streptococcus pneumoniae*	Isolated strains from sputum	N	1
65	*Streptococcus suis*	Isolated strains from feces	N	1
66	*Vibrio vulnificus*	Isolated strains (U)	N	0

^a^ MCDA assay result: detected using the colorimetric indicator method. P, positive for the MCDA assay; N negative.

^b^ U: unknown.

### The clinic samples evaluation for the MCDA based identification of *K*. *pneumoniae*

In order to evaluate the practical application of the *K*. *pneumoniae*-MCDA-LFB assay, we assessed its LOD for *K*. *pneumoniae* strains in sputum samples. The MCDA-LFB assay was able to generate positive results when the number of *K*. *pneumoniae* strains in the sputum samples was more than 2.4 × 10^3^ CFU/ml (24 CFU per reaction) (S2 Fig). It was 100 times more sensitive than the PCR methods, the LOD of which was 2.4 × 10^5^ CFU/ml (2400 CFU per reaction).

Furthermore, DNA templates of 100 sputum samples from the hospitalized patients underwent the *K*. *pneumoniae*-MCDA-LFB test, with 30 sputum samples being identified positive for this pathogen. The sputum samples being cultured positive for other bacteria (including *Acinetobacter baumannii*, *Staphylococcus aureus*, *Pseudomonas aeruginosa* and *Staphylococcus hominis*) were negative for the *K*. *pneumoniae*-MCDA-LFB assay. The results of the *K*. *pneumoniae*-MCDA tests were in complete agreement with that of the culture method. In contrast, the PCR assay only identified 25 positive samples (Table 3). Thus, the sensitivity and specificity for *K*. *pneumoniae*-MCDA-LFB assay were both 100%, while they were 83.3% and 100% for PCR, respectively (S3 Fig).

**Table 3:** Clinical application of the *K*. *pneumoniae*-MCDA-LFB assay in sputum samples.

		Reference method	
Method	Result	Positive	Negative
**MCDA-LFB**	Positive	30	0
	Negative	0	70
**PCR**	Positive	25	0
	Negative	5	70

## Discussion

*K*. *pneumoniae* is a well-recognized reason for the nosocomial infections [1]. Early and rapid diagnosis of this pathogen in the clinical samples of patients like sputum, blood, urine and other fluids, will help control the development of infections, which is utmost important for the critical patients. Many *K*. *pneumoniae* outbreaks were found to be associated with the environmental or equipment contaminations, e.g. in the bronchoscopy, endoscopy, and water sink in the ICU [3,4,7,18]. A rapid and simple detection method with high sensitivity will be useful for the rapid identification of *K*. *pneumoniae* infections and the surveillance of this pathogen in the healthcare environment. As far as we know, reports about rapid detection assays of *Klebsiella pneumoniae* is few, all of which were based on the technique of loop mediated isothermal amplification (LAMP) [16,19]. There were 2 to 2.5 pairs of sequences working on the target sequence with the LAMP method, making it more rapid and sensitive than the normal PCR method [19,20]. It has been reported that by adding additional primers for the isothermal amplification, the sensitivity can increased and the amplification time can be reduced [21,22]. Thus, we introduced the technique of MCDA, which has five pairs of primers working on the target sequence at the same time, for the rapid detection of *K*. *pneumoniae* targeting the *rcs*A gene [8]. *RcsA* gene, which regulates the capsule production of *K*. *pneumoniae*, had been reported to be one specific gene for this pathogen [16,23]. The MCDA detection of *K*. *pneumoniae* could be completed within 40 minutes at 65°C, which is similar to the other MCDA-based assays [15,17].

Besides, this study introduced the simple, rapid and objective gold-nanoparticle-based LFB for the results reading. The same with Wang *et al*.’s results, all the *K*. *pneumoniae* positive MCDA products were successfully identified with LFB, with two red lines occurred within 2 minutes [9]. By identifying the amplification products labeled with FITC and biotin, the results recognized by LFB are relatively more objective and reliable than that of turbidimeters and colorimetric indicators [9]. Although the gel electrophoresis method could distinguish the specific amplification and non-specific amplification, it is laborious and equipment demanding. Considering the high work load of diagnosis and examination in the clinical laboratory, LFB will be one efficient candidate for the results reading of either normal PCR or isothermal amplification approaches.

The detection limit of the *K*. *pneumoniae*-MCDA assay was determined as 100 fg. Other MCDA based studies also found that the threshold of detection is ranging from 1 pg to 10 fg [9,17]. In fact, the isothermal amplification assays can be more sensitive by removing the carryover contamination and non-specific amplification, with the further lower concentration of DNA being recognized after longer amplification time [22].

The 100% positivity of the 30 clinical-source *K*. *pneumoniae* strains and the 100% negativity of the 35 non-*K*. *pneumoniae* strains indicated that the MCDA primers based on *rcsA* gene were specific to *K*. *pneumoniae*. However, carryover contamination is a great concern for genome template based identification assays, especially for the isothermal amplification methods with high concentration of primers. We noted examples of false positive results occurring when the amplification was prolonged for more than 40 minutes. Limiting the amplification time has been suggested by Wang *et al*. to reduce the carryover amplification [22]. According to the turbidity graph of sensitivity (Fig 4A**)**, the amplification of 100 fg DNA reached the positive threshold of 0.1 after 25 minutes. Thus, we suggest the amplification time of no more than 40 minutes, in order to reduce the interference of carryover amplification and non-specific amplification. And it was recently found that the carryover contamination can be removed by adding the Antarctic thermolabile uracil-DNA-glycosylase into the reaction system before amplification [22].

We evaluated the LOD of the MCDA-LFB in clinical samples by artificially contaminating the *K*. *pneumoniae*-negative sputum sample with the ATCC2146 of different concentrations. Using the DNA of the sputum samples as templates, the LOD of the *K*. *pneumoniae*-MCDA-LFB assay was 24 CFU per reaction, which was 100-fold more sensitive than the PCR method. Other reports also showed that the MCDA-based assay was more sensitive than the PCR method in clinical or food samples [9,24]. The sensitivity of the MCDA assay did not decrease in mixture of other non-*K*. *pneumoniae* strains of high concentration. Moreover, we evaluated the practical applicability of MCDA for *K*. *pneumoniae* in 100 clinical sputum samples. All of the samples positive for the *K*. *pneumoniae* with culture method were successfully identified with the MCDA assay. While only 25 samples were recognized with the PCR method. This may be attributed to the lower sensitivity of PCR method in sputum samples. The sputum samples being cultured positive for other pathogenic bacteria were negative for the *K*. *pneumoniae*-MCDA-LFB assay, in agreement with the specificity evaluation results. The detection limit of the MCDA-LFB assay did not decrease when the K. pneumoniae strains were in mixture of the other strains. Thus, the *K*. *pneumoniae-*MCDA-LFB assay can be used as a candidate for the rapid identification of this pathogen in the original samples like sputum. Traditional culture requires for at least two days and is equipment dependent, isothermal amplification methods such (loop-mediated isothermal amplification) LAMP has been applied in the identification of infection in the clinical samples [25,26]. The results of this study suggested that the MCDA method could be used to identify *K*. *pneumoniae* infections.

## Conclusion

We developed a rapid and simple isothermal amplification and identification assay for *K*. *pneumoniae* based on the proof-of-concept MCDA and LFB. DNA templates of the *K*. *pneumoniae* strains and the non-*K*. *pneumoniae* strains were successfully recognized with MCDA. The detection limit of this assay is 100 fg which can be recognized after 25 minutes of isothermal reaction at 65°C. The MCDA-LFB assay could efficiently identify *K*. *pneumoniae* in clinical samples such as sputum.

## Supporting information

S1 Table PCR primers for *rcsA* gene to detect *K*. *pneumoniae*. (DOCX)

S1 Fig Sensitivity of the MCDA-LFB assay to detect *K*. *pneumoniae* in mixtures with other non-*K*. *pneumoniae* strains. LFBs of 1 to 8 refer to MCDA reactions containing different DNA concentrations of *K*. *pneumoniae* (10 ng, 10 pg, 1 pg, 100 fg, 10 fg, 1 fg, 0.1 fg, and the blank control) and 67.5 ng of *Staphylococcus aureus* per reaction (**A**), 130.0 ng of *Salmonella typhii* per reaction (**B**), 55.3 ng of *Acinetobacter baumannii* per reaction (**C**), and 82.2 ng of *Streptococcus pneumoniae* per reaction (**D**). The detection limit of MCDA-LFB assay was 100 fg, showing both the test line and the control line on the LFBs. MCDA, multiple cross displacement amplification; LFB, gold nanoparticle lateral flow biosensor.
(TIF)

S2 Fig Detection limit of the *K*. *pneumoniae* MCDA assay in artificially contaminated sputum samples. MCDA reactions containing different concentrations of *K*. *pneumoniae* reference strain ATCC2146 (24, 000 CFU, 2, 400 CFU, 240 CFU, 24 CFU, and 2.4 CFU per reaction) using *Staphylococcus aureus* and *Salmonella typhii* as negative controls (NC) and distilled water as blank control (BC). The LOD in sputum samples was 24 CFU per reaction according to the real-time turbidimeter (**A**). The amplification products were electrophoresed in 2% agarose gel, with the reactions containing 24,000 CFU to 24 CFU of *K*. *pneumoniae* showing ladder bands (**B**). This was in agreement with the results of the colorimetric indicators and LFBs, the positive reactions of which showed a blue color and two red bands on the LFBs, respectively (**C**, **D**). MCDA, multiple cross displacement amplification; LFB, gold nanoparticle lateral flow biosensor.
(TIF)

S3 Fig The ROC curve analysis for the MCDA-LFB assay and PCR in 100 clinical sputum samples. The sensitivity and specificity for K. pneumoniae-MCDA-LFB assay (solid line) were both 100%, while they were 83.3% and 100% for PCR (dash line). ROC, receiver-operating characteristic; MCDA, multiple cross displacement amplification; LFB, gold nanoparticle lateral flow biosensor.
(TIF)
