# Supplementary material for: Isothermal amplification and rapid detection of Klebsiella pneumoniae based on the multiple cross displacement amplification (MCDA) and gold nanoparticle lateral flow biosensor (LFB)
Source: PLoS One. 2018 Oct 1;13(10):e0204332. doi: 10.1371/journal.pone.0204332 (PMC6166938; doi:10.1371/journal.pone.0204332)
Supplement: S1 Table — (DOCX) [file pone.0204332.s001.docx]

S1 Table. PCR primers for *rcsA* gene.

| Gene | Sequence (5'->3') | Template strand | Product length | Annealing temperature |
| --- | --- | --- | --- | --- |
| *rcsA* | Forward primer | TGTGCAGCTATACCCGGTTG | 564 | 60^o^C |
|  | Reverse primer | CGGTCAGCCGAACGATATGA |  |  |
