# Supplementary figures and images for: Isothermal amplification and rapid detection of Klebsiella pneumoniae based on the multiple cross displacement amplification (MCDA) and gold nanoparticle lateral flow biosensor (LFB)
Source: PLoS One. 2018 Oct 1;13(10):e0204332. doi: 10.1371/journal.pone.0204332 (PMC6166938; doi:10.1371/journal.pone.0204332)

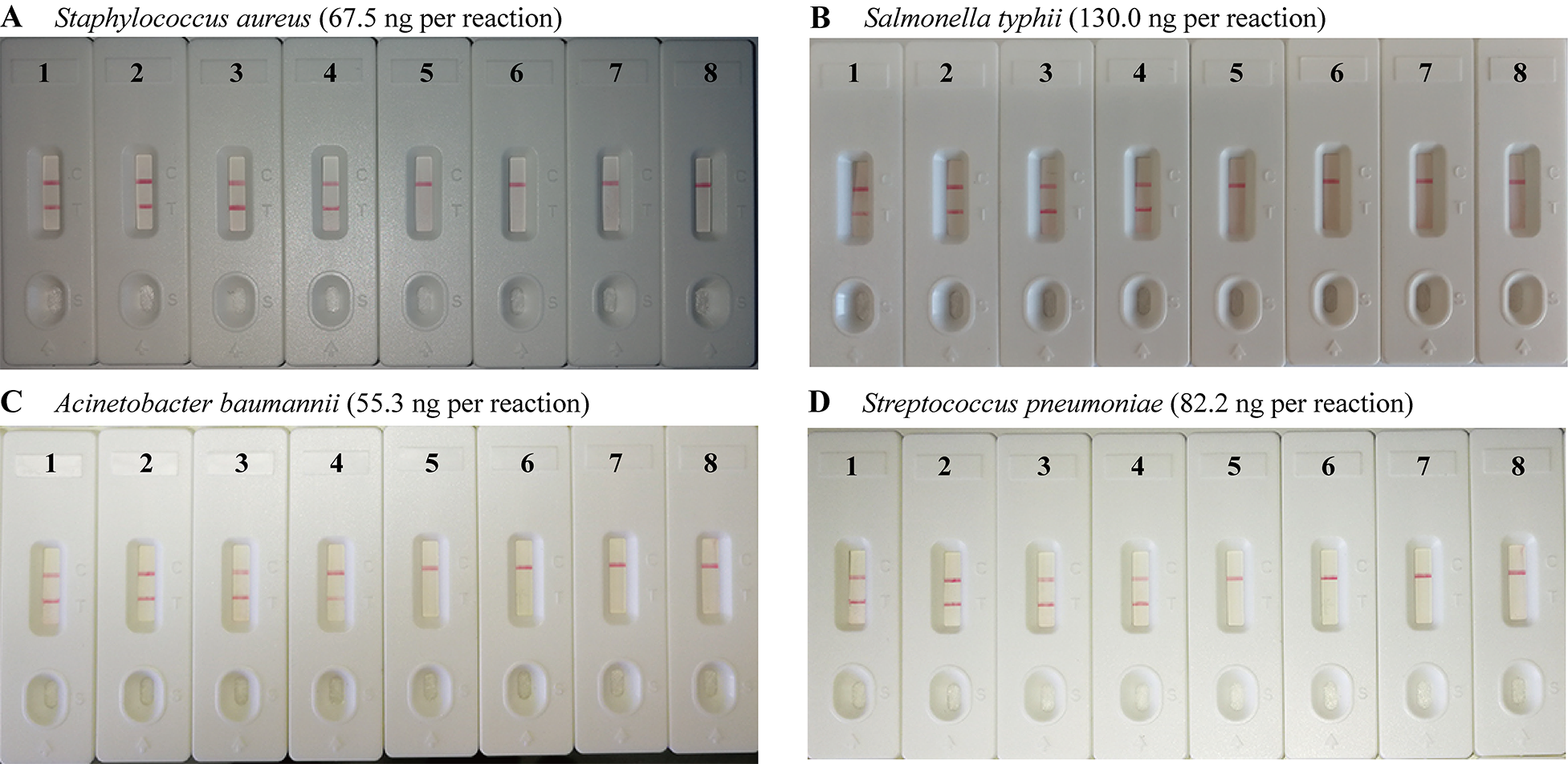

Supplement: S1 Fig — LFBs of 1 to 8 refer to MCDA reactions containing different DNA concentrations of K. pneumoniae (10 ng, 10 pg, 1 pg, 100 fg, 10 fg, 1 fg, 0.1 fg, and the blank control) and 67.5 ng of Staphylococcus aureus per reaction (A), 130.0 ng of Salmonella typhii per reaction (B), 55.3 ng of Acinetobacter baumannii per reaction (C), and 82.2 ng of Streptococcus pneumoniae per reaction (D). The detection limit of MCDA-LFB assay was 100 fg, showing both the test line and the control line on the LFBs. MCDA, multiple cross displacement amplification; LFB, gold nanoparticle lateral flow biosensor. (TIF) [file pone.0204332.s002.tif]

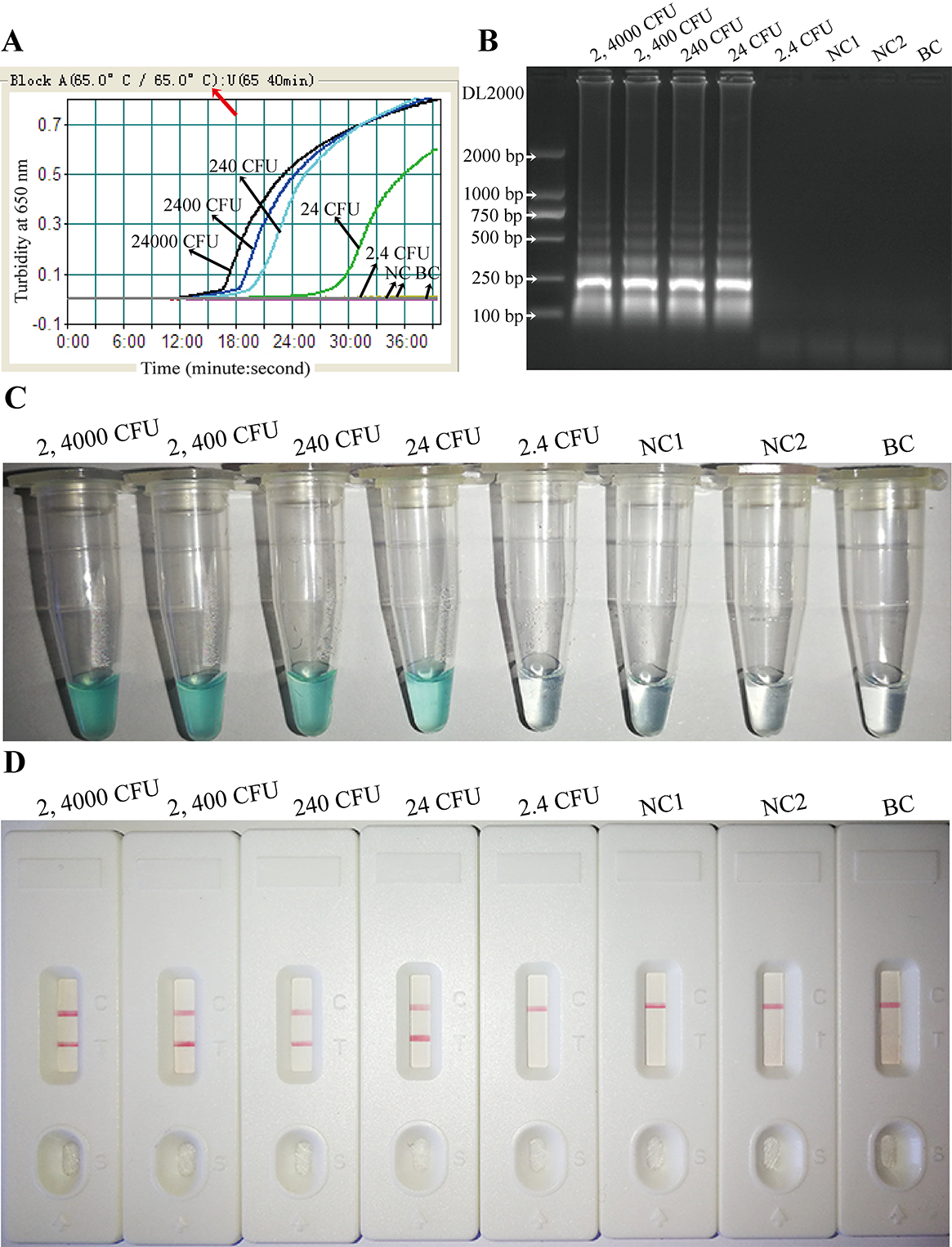

Supplement: S2 Fig — MCDA reactions containing different concentrations of K. pneumoniae reference strain ATCC2146 (24, 000 CFU, 2, 400 CFU, 240 CFU, 24 CFU, and 2.4 CFU per reaction) using Staphylococcus aureus and Salmonella typhii as negative controls (NC) and distilled water as blank control (BC). The LOD in sputum samples was 24 CFU per reaction according to the real-time turbidimeter (A). The amplification products were electrophoresed in 2% agarose gel, with the reactions containing 24,000 CFU to 24 CFU of K. pneumoniae showing ladder bands (B). This was in agreement with the results of the colorimetric indicators and LFBs, the positive reactions of which showed a blue color and two red bands on the LFBs, respectively (C, D). MCDA, multiple cross displacement amplification; LFB, gold nanoparticle lateral flow biosensor. (TIF) [file pone.0204332.s003.tif]

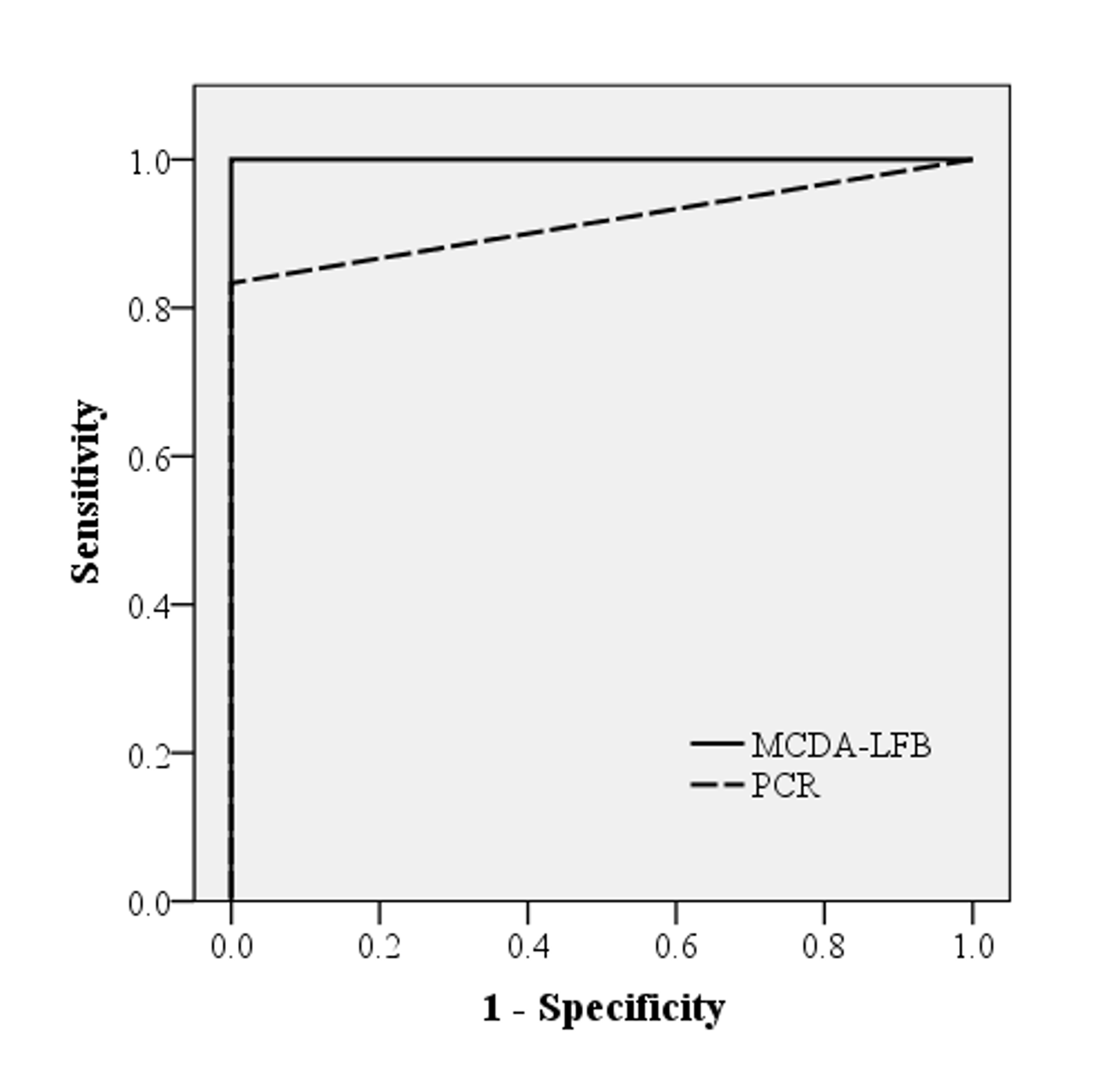

Supplement: S3 Fig — The sensitivity and specificity for K. pneumoniae-MCDA-LFB assay (solid line) were both 100%, while they were 83.3% and 100% for PCR (dash line). ROC, receiver-operating characteristic; MCDA, multiple cross displacement amplification; LFB, gold nanoparticle lateral flow biosensor. (TIF) [file pone.0204332.s004.tif]
